# Insights from a Bibliometrics-Based Analysis of Publishing and Research Trends on Cerium Oxide from 1990 to 2020

**DOI:** 10.3390/ijms24032048

**Published:** 2023-01-20

**Authors:** Charlotte L. Fleming, Jessie Wong, Mojtaba Golzan, Cindy Gunawan, Kristine C. McGrath

**Affiliations:** 1 School of Life Sciences, Faculty of Science, University of Technology Sydney, Sydney, NSW 2007, Australia; 2 Vision Science Group, Graduate School of Health, University of Technology Sydney, Sydney, NSW 2007, Australia; 3 Australian Institute for Microbiology and Infection, University of Technology Sydney, Sydney, NSW 2007, Australia

**Keywords:** nanoceria, nanoparticles, composition, applications, biomedicine, review

## Abstract

The purpose of this study is to evaluate the literature for research trends on cerium oxide from 1990 to 2020 and identify gaps in knowledge in the emerging application(s) of CeONP. Bibliometric methods were used to identify themes in database searches from PubMed, Scopus and Web of Science Core Collection using SWIFT-Review, VOSviewer and SciMAT software programs. A systematic review was completed on published cerium oxide literature extracted from the Scopus database (*n* = 17,115), identifying themes relevant to its industrial, environmental and biomedical applications. A total of 172 publications were included in the systematic analysis and categorized into four time periods with research themes identified; “doping additives” (*n* = 5, 1990–1997), “catalysts” (*n* = 32, 1998–2005), “reactive oxygen species” (*n* = 66, 2006–2013) and “pathology” (*n* = 69, 2014–2020). China and the USA showed the highest number of citations and publications for cerium oxide research from 1990 to 2020. Longitudinal analysis showed CeONP has been extensively used for various applications due to its catalytic properties. In conclusion, this study showed the trend in research in CeONP over the past three decades with advancements in nanoparticle engineering like doping, and more recently surface modification or functionalization to further enhanced its antioxidant abilities. As a result of recent nanoparticle engineering developments, research into CeONP biological effects have highlighted its therapeutic potential for a range of human pathologies such as Alzheimer’s disease. Whilst research over the past three decades show the versatility of cerium oxide in industrial and environmental applications, there are still research opportunities to investigate the potential beneficial effects of CeONP in its application(s) on human health.

## 1. Introduction

Cerium oxide (CeO_2_), also referred to as nanoceria, is a well-known rare earth element of moderate abundance, found in the lanthanide group in the periodic table [1]. The unique structural properties of CeO_2_ allow its electron configuration to alter, such that it can exist in both the trivalent Ce^3+^ (Ce_2_O_3_) reduced state and the stable tetravalent Ce^4+^ (CeO_2_) oxidized state [1]. This property enhances oxygen storage and release, resulting in its ability to regenerate based on redox cycling in its immediate environment [2,3,4]. Cerium (Ce) when added to oxygen (O_2_) in nanoparticle formulation (CeONPs) adopts a cubic crystalline structure, akin to a fluorite type structure, enabling the rapid diffusion of oxygen as a function of the number of oxygen vacancies [1,4].

CeONP can be comprised of different nanostructure size, shape, morphology, and composition and have been shown to have strong antioxidant mimetic properties, reflecting the behaviour of enzymes like superoxide dismutase (SOD), catalase, and peroxidase, targeting and scavenging reactive oxygen species (ROS) and reactive nitrite species (RNS) [5]. In addition to the strong antioxidant property CeONP has, it has also been shown to have anti-inflammatory, antibacterial and anti-cancer activity [6,7,8]. Given the various potential application demonstrated for CeONPs, numerous literatures on the potential of CeONP has been published in recent years.

Bibliometric analysis is a branch of scientific computer-assisted review analysis that integrates mathematical and statistical approaches to evaluate academic literature in a specific field [9,10]. Analysis using bibliometric tools have been applied to evaluate research that have been undertaken in specific fields of science to provide a global perspective on how research changes overtime, how they relate to one another, research contribution from different countries and subsequently highlight trends surfacing from the various publications over time [9,10,11]. Bibliometrics studies have been conducted on both magnetite and titanium dioxide nanoparticles, to identify particular hotspots in research, but also highlight the gaps in knowledge about these nanoparticles [11,12]. To the best of our knowledge, no bibliometric studies have been conducted on cerium oxide over the past three decades. The aim of this study was to conduct a bibliometric analysis on studies on cerium oxide published over thirty years from 1990 to 2020 to identify publication trends, the most productive countries in cerium oxide research and research hotspots over the years. This paper will serve as a reference to aid in current and future development of research on cerium oxide.

## 2. Results

The results from the three databases using the search terms “cerium oxide OR ceria OR nanoceria OR nano ceria” yielded the highest number of publications regardless of the database used. A large difference in the number of publications returned between the databases was noted, with WoS returning ~238% and ~180% more papers compared to PubMed and Scopus, respectively (Table 1). The search term “cerium oxide OR ceria OR nanoceria OR nano ceria AND pathology” returned the smallest number of publications with ~57% less for WoS, ~87% less for PubMed and ~99% less Scopus.

### 2.1. Topic Modelling

**Figure 1:**
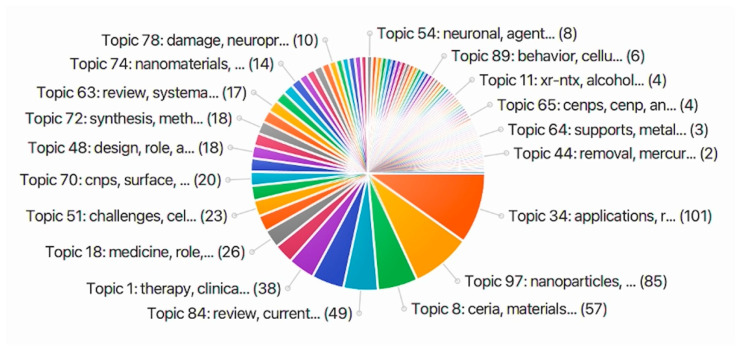
The top 19 topic models formulated from the dataset collected from PubMed (129 publications) with the SWIFT-Review software, using the search term “cerium oxide OR ceria OR nanoceria OR nano ceria”. This search was refined to review, clinical trials, meta-analysis, and research articles. Accessed on the 17 September 2020.

To identify the focus and applications of CeONPs studies conducted from 1990 to 2020, the search “cerium oxide OR ceria OR nanoceria OR nano ceria” was completed using the PubMed database. The database yielded 129 publications which was imported into SWIFT-Review software. The SWIFT-Review software analysed the articles and segregated them based on keywords, where they were then categorized into topic models and organized in ranking order. The SWIFT-Review software found 100 topic models with an overview of the top 19 topic models shown in Figure 1. Analysis of these top models identified research were mainly focus on the biomedical applications of CeONPs, focusing its oxidative properties and surface modifications (Table 2).

### 2.2. Bibliometric Analysis

**Figure 2:**
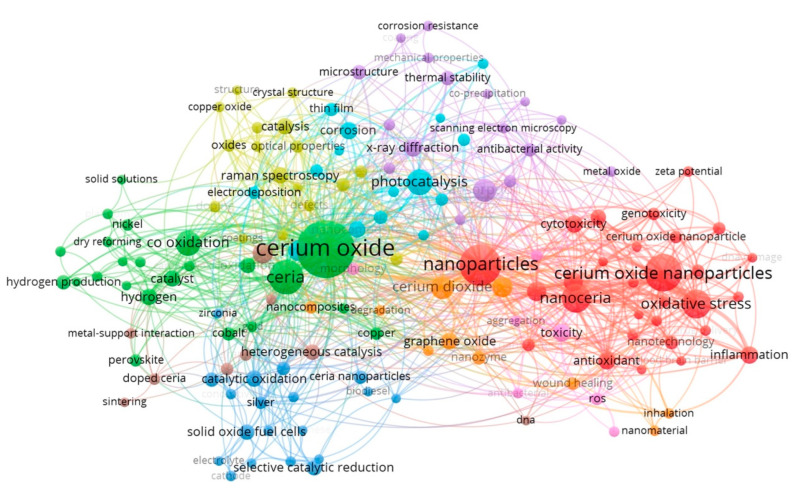
Network visualization map showing nine color-coded clusters produced using the VOSviewer and the WoS Database with the search “cerium oxide OR ceria OR nanoceria OR nano ceria”. The network analysis from 7862 publications and 144 author key words, from 1990 to 2020. The colours represent the clusters of keywords presented in Table 3. Access on the 17 September 2020.

To examine the broad development of literature in the research associated with CeONPs, the search “cerium oxide OR ceria OR nanoceria OR nano ceria” in the years 1990–2020 was implemented in the WoS database, yielding 7862 publications. To create a visualization of the co-occurrence of all keyword terms, the extracted dataset (title, abstract and author keywords) was imported into the VOSviewer Software (www.vosviewer.com), Universiteit Leiden, Leiden, Netherlands, Version 1.6.15). The main characteristics obtained from an analysis of the co-occurrence of keywords included the frequency and proximity of similar words. The keywords were further refined to a minimum of 20 occurrences, yielding 144 keywords, then designated by VOSviewer into 9 main cluster groups (Table 3). Each cluster represents major themes of CeONPs studies between 1990–2020. Important findings within each cluster are established with a lay description (Table 3) and a corresponding visualization map with the clusters coded by colour presented in Figure 2. Cluster 1 (red) refers to the biocompatibility and antioxidant properties of CeONPs in biomedicine. Some of the clusters are closely associated; clusters 2 and 4 (green and yellow, respectively) highlight the different doping additives, as well as changes in the nanostructure to increase efficiency in various applications. Clusters 3 (blue), 5 (purple) and 6 (aqua) all refer to CeONP application in environmental remediation and industrial implementation as biofuels. Cluster 7 (orange) refers to CeONPs as additives to biofuels and as a potential therapy for wound healing. Lastly, clusters 8 (brown) and 9 (pink), mention the oxygen defects (vacancies) on the surface and antioxidant activity of CeONPs. This analysis identified nanoparticle engineering enhancements as well as specific fields of nanoparticle use (i.e., environmental remediation), which are further investigated.

### 2.3. SciMAT

**Figure 3:**
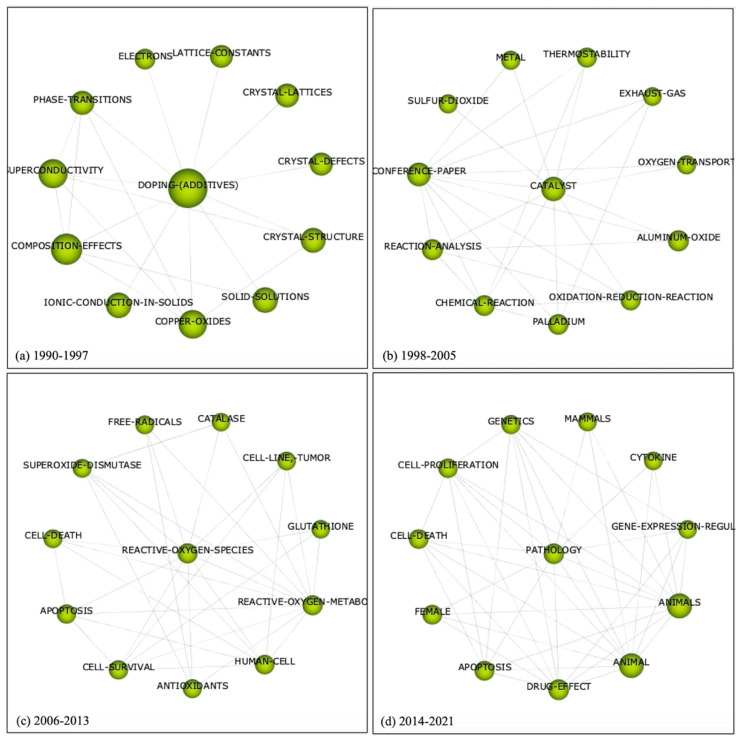
Main themes in the cerium oxide related publications from the Scopus database, over four time periods identified using SciMAT software (**a**) 1990–1997 (*n* = 1047); (**b**) 1998–2005 (*n* = 2937); (**c**) 2006–2013 (*n* = 7858); (**d**) 2014–2020 (*n* = 5273). The figure demonstrates the development of cerium oxide investigation and the links in terms of the major themes, identified for each period. Accessed on the 9 October 2020.

A longitudinal bibliometric study was performed using the SciMAT software from the Scopus dataset (17,115 publications). From 1990 to 1997, the results show a clear trajectory of research interest on CeO_2_ with the term “doping additives” emerging as the main theme in this time period (*n* = 1047). Studies in this time period were focused on enhancing the properties of CeO_2_ through incorporation of additives (e.g., doping, coating, nanocomposites and hybrid nanostructures) and morphological alterations as indicated by the terms “crystal defects” and “crystal lattice (Figure 3a). The term “catalyst” was highlighted as a main theme in 1998–2005 (*n* = 2937) associated with the terms “thermostability”, “oxygen transport” and “exhaust gas” suggesting its catalytic role as a potential air pollutant adsorber or a biofuel (Figure 3b). In the period of 2006–2013 (*n* = 7858 publications), “reactive oxygen species” was identified as the main theme, with terms like “cell survival”, “cell death”, “free radicals”, “catalase” and “glutathione”, indicating the research focused on antioxidant properties on cellular models (Figure 3c). The final period between 2014–2020 (*n* = 5273 publications) indicated “pathology” as a main theme, with “apoptosis”, “cell proliferation” and “drug effect” identified as associated terms suggesting that research in this period focused on CeONPs and its effects on gene expression and cell proliferation as a biomedicine (Figure 3d).

### 2.4. Systematic Review of Literature

**Figure 4:**
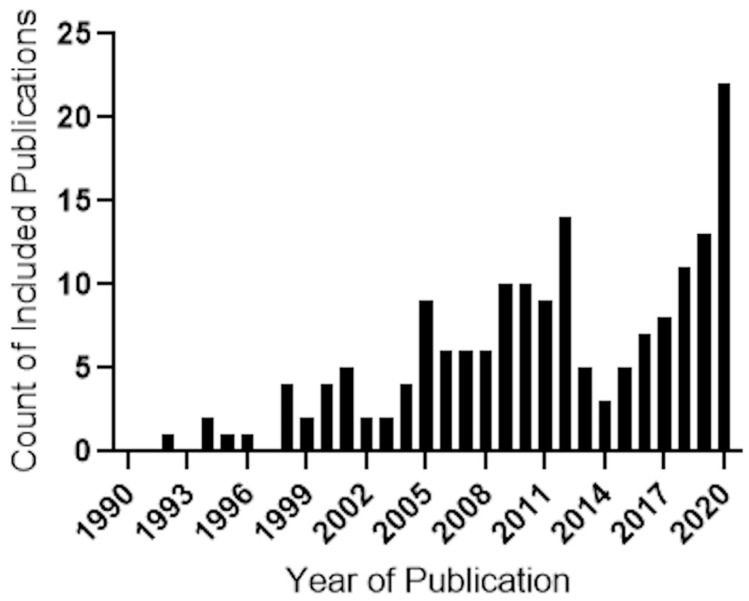
Frequency histogram of 172 publications, derived from a systematic search of cerium oxide literature, conducted in the Scopus database for search terms: “cerium oxide OR ceria OR nanoceria OR nano ceria” AND doping-additives/catalysts/reactive oxygen species/pathology, showing the surge of publication activity across all four themes between 2019–2020. The year of 2020 demonstrating the highest number of publications. Accessed on 17 September 2020.

A refined search on the Scopus database was then completed to provide a systematic analysis of the literature from each theme identified from the SciMAT software. A total of 172 publications, refined by a citation ratio of greater than 6 (peer-acknowledged quality threshold), were included in this study. Figure 4 shows the total publications that met the citation criteria; 5 publications were from 1990 to 1997 (“doping additives”), 32 publications from 1998 to 2005 (“catalyst”), 66 publications from 2006 to 2013 (“reactive oxygen species”), and 69 publications from 2014 to 2020 (“pathology”).

#### 2.4.1. Time Period 1990–1997—“*Cerium Oxide* and Doping-(Additives)”

SciMAT extracted five publications from this period meeting the citation ratio of above 6, focusing on the combined environmental and industrial applications of CeONPs. To enhance CeONPs ionic conductivity for electrolyte fuel cell efficiency, one study doped the CeONPs with zirconia, samaria and gadolinia which resulted in increased oxygen vacancies (or oxygen defects) on its surface thereby making it more reactive [13]. Samaria and gadolinia-doped CeONPs exhibit high electrical conductivity due to the close ionic radii of Sm^3+^ Gd^3+^ to that of Ce^4+^ [13]. The reduction of ceria electrolyte at the fuel side could be suppressed by doping with a thin film of stabilized zirconia on the ceria surface [13].

While the theme of this time period is “doping-additives” of CeONPs, the remaining studies included in this analysis focused mainly on the morphological enhancements of CeONPs due to the FCR threshold implemented. Two publications focused on using bare CeONPs to eliminate contaminants in automobile gas exhaust [14,15]. Bare CeONPs have less oxygen vacancies thereby increasing nanoparticle stability suitable for interactions between small molecules, e.g., hydrogen, carbon dioxide, oxygen and nitric oxide [14,15]. Another publication in this period focused on the structural properties of CeONPs, finding that morphology and surface characteristics greatly influence the behaviour of the nanoparticles, e.g., lattice structure with increased surface area exposed have more oxygen vacancies with a less compact nanostructure but increased reactivity [16]. The last publication in this time focused on CeONPs as an effective industrial catalyst by increasing the efficient of the WGS reaction, which was achieved at low temperature thereby conserving energy [17]. The research on CeONPs in this period highlight its potential as an environmental/industrial fuel cell and energy converting catalytic alternative to fossil fuel sources, which is a topic further investigated in successive time periods.

#### 2.4.2. Time Period 1998–2005—“*Cerium Oxide* and Catalyst”

In this time-period, 32 publications met the citation ratio criteria, overlapping themes from the previous period including the environmental/industrial applications with advancements (e.g., additives to CeONPs) in renewable energy and as a potential biomedicine in its pure form.

The environmental/industrial applications of CeONPs in this period, make up most of the publications (*n* = 23) and extend on the previous period with different additives and structural changes. For example, the nanocomposite structure incorporating nanosized particles into a matrix of standard material thus resulting in improved strength, electrical and thermal properties [18]. Many publications focused on CeONPs as renewable energy sources to replace combustion fuel sources (e.g., natural gas, diesel fuel, biodiesel blends, petroleum and coal) [19]. For example, one study used poly-alkene doped CeONPs in a crystalline form with a porous surface thereby increasing the number of surface and oxygen vacancies to enhance the thermo-catalytic stability in solar cells in replacement organic dyes [20]. Gadolinia-doped CeONPs, as a solid fuel electrolyte, was found to have high stability at reduced oxygen pressures improving fuel cell performance while maintaining textural and mechanical integrity, increasing the electrolyte composition therefore making it a promising recyclable renewable source for turbine power [21,22]. Other studies highlighted the use of copper and nickel loaded CeONPs, doped with lanthanum in a hybrid nanocrystalline structure (i.e., hybrid—combination of doping, loading, surface functionalized or nanocomposite additives) [23]. These hybrid structures were highly dispersed due to the doping and loaded component interactions which enhances their catalytic activity through the increased oxygen vacancies of the added components thereby increasing WGS reaction efficiency [23]. CeONPs have also been used as effective pollutant adsorbers, decontaminating wastewater and sediment following the use of additives such as magnesium oxide/aluminium oxide/titania oxide to generate nanocomposites. Modification of the CeONPs for the removal of heavy metals and organic compounds indicated the redox potential to adsorb the contaminants was increased, suggesting the additives and nanostructure enhanced these properties [24,25,26,27,28]. CeONPs nanocomposites have also been investigated as highly efficient catalysts (e.g., copper, zirconia, nickel, aluminium/CeONPs nanocomposites) for methane oxidation (methane is a potent greenhouse gas in the atmosphere and needs to be broken down by combustion, producing heat and therefore making it a useful fuel source) [24,25,29,30]. These nanocomposite structures are ideal for methane oxidation due to catalytic nature of CeO_2_, achievable at lower temperatures, hence conserving more energy. Platinum and gold/CeONPs nanocomposites in a crystal lattice fluorite structure showed increased reactivity and reducibility for a more efficient catalyst in the WGS reaction as the temperature can be lowered allowing for enhanced energy to be conserved [31,32,33,34,35]. The morphology of CeONPs has been studied in this period (*n* = 7) to enhance the properties for a specific purpose, with nano-polyhedral, nano-rods, and nanotubes shown to have increased oxidative properties compared to those of the nanosphere [36]. The crystal plane structure of CeO_2_ nano-rods was found to have higher oxidation activity compared to crystal lattice structure. As more oxygen vacancies are exposed, the structure becomes more reactive and therefore superior fuel cells and environmental remediators [37,38]. A reoccurring theme for environmental/industrial catalysts in this period highlights the manipulation in manufacturing nanotechnology to increase oxygen vacancies on the surface of the nanostructures which improves the catalytic activity. This is further explored in the following period.

The biomedical applications in this period (*n* = 2) highlighted CeONPs as potential cancer therapies. One study investigated the cellular uptake of polymer micelle coated CeONPs in human lung fibroblasts in vitro models (ATCC and MRC-9 cell lines). These nanostructures showed increased adsorption, agglomeration, dispersion, and retention time compared to bare CeONPs therefore proving to be a superior cancer therapy [39]. The second demonstrated that bare CeONPs in a crystal lattice structure showed differential effects with 99% protection against radiation-induced cell death in human breast carcinoma epithelial in vitro model (MCF-7 cell line) whilst no protection was conferred for the normal human breast epithelial in vitro model (CRL8798 cell line) [40]. These studies provide a good foundation into the successive periods of CeONPs as a biomedicine, with its potential as a cancer drug delivery agent and in chemotherapy radiation.

#### 2.4.3. Time Period 2006 to 2013—“*Cerium Oxide* and Reactive Oxygen Species”

A total of 66 publications met the citation criteria in this period, with overlapping themes from the previous period: CeONPs as an effective environmental/industrial renewable resource for fuel cells and its biomedical application as a cancer therapy. The environmental/industrial uses of CeONPs in this period (*n* = 47) highlight the manufacturing manipulations focused on increasing the amount of oxygen vacancies through nanoparticle engineering in adjusting the additives and morphology of the nanostructures. Two studies focused on the effectiveness of CeONPs as an electrolyte fuel cell, with studies assessing samaria- and carbon-based polymer doped CeONP hybrids to increase oxygen vacancies and induce higher energy conversion rates that subsequently improved efficiency, coinciding with low emissions [41,42]. Studies in this period have again highlighted CeONPs as solid electrolyte fuel cells, however in the lattice fluorite structure. This structure improved ionic conductivity and overall efficiency when doped with lower charged cations like zirconia oxide, aluminium oxide, and palladium oxide due to the increased oxygen vacancies on the surface and the interactions between these components [43,44]. Of particular significance in this period, was the platinum oxide, titania oxide, copper oxide, and particularly gold oxides/CeONPs nanocomposites, extensively investigated for further improved efficiency in the WGS reaction [45,46,47,48,49,50,51,52,53]. Adding heterogenous “- oxide” nanoparticles to the CeONP nanocomposite further enhanced the formation of oxygen vacancies on the surface, which increased the catalytic activity of these nanostructures [45,46,47,48,49,50,51,52,53]. Various research inquiries studied CeONPs nanocomposites (e.g., platinum and zirconia/CeONPs) for automotive exhaust gas remediation. These studies found that the nanowire structures, with increased surface presence of oxygen vacancies, were associated with the highest catalytic activities, as well as recyclability of CeONPs [54,55,56,57,58]. These studies showcased innovations in nanostructure engineering, to increase the surface presence of oxygen vacancies, and in turn, enhances the nanostructures catalytic activities. The presence of oxygen vacancies enable CeONPs to readily scavenge free radicals present in pollutants, such as superoxide (O_2_^−^), hydroxide (OH^−^), hydroxyl (OH) and hydrogen peroxide (H_2_O_2_), nitric oxide (NO) and peroxynitrite (ONOO^−^), thereby beneficial in environmental remediation [59]. This ROS scavenging capability also prompted the biomedical application of CeONPs.

The biomedical research on CeONPs (*n* = 15) in this period further investigated the potential of CeONPs and exploited the advancements in nanoparticle engineering additives like doping, surface modification and nanocomposites. CeONPs have been found to be protective against ischemic stroke in a cardiac progenitor in vitro model showing no toxicity, in fact increasing cell viability and decreasing apoptosis compared to copper and zinc nanoparticles [60,61,62]. CeONPs have also been used as probes in bioanalysis and diagnostic tests, in the form of a bioactive sensing paper for glucose and H_2_O_2_ testing to replace the use of organic dyes, measuring multiple cycles [63]. Studies have shown that CeONPs have antioxidant catalytic properties, having the capacity to mimic superoxide dismutase (SOD), specifically converting superoxide to oxygen and hydrogen peroxide with high specificity and efficiency, highlighting its ability to scavenge ROS and RNS and therefore an effective antioxidant [64,65,66]. The term “doping” consists of the insertion of a new specific ion into a crystal lattice structure, where “coating” consists of a thin film applied to the surface, encapsulating a nanomaterial [67]. Conjugation of CeONPs with polymers (e.g., polyacrylic acid, animated poly(acrylic acid), dextran-coated) were indicated to increase CeO_2_ oxidase-like activity, as well as enhancing the nanoparticles cell targeting activities, and in turn, their cellular uptake in in vitro models (lung carcinoma—A549, cardiac myocytes—H9c2, embryonic kidney—HEK293 and breast carcinoma—MCF-7 cell lines) [68,69]. Dextran-conjugated CeONPs were found to exhibit protective effects on human dermal fibroblast (HDF) cell line, being correlated to the particle ROS scavenging ability [70]. Another study by Kim and colleagues showed polyethylene glycol (PEG)-conjugated CeONPs protected against ROS-induced cell death in vitro (using ovarian hamster CHO-K1 cells) and subsequently observed protection against ischemic induced-apoptotic cell death in the brain in an in vivo model of ischemic stroke [71]. Similarly, Asati and colleagues investigated yttrium oxide/CeONP nanocomposites on a nerve cell model (HT22 cell line) and observed neuroprotective effects of the nanoparticles, with a lowered oxidative stress and cell toxicity [72]. These studies show the broad range of biomedical applications and disease pathologies, which CeONPs have been implicated for, highlighting the nanotechnology advancements that improve the biocompatibility of the nanostructures (e.g., ROS scavenging ability to reduce oxidative stress, indicated increased circulation time and cellular uptake). These studies have directly influenced the biomedical applications of CeONPs in the following period (2014–2020).

Four publications in this period reported the potential toxicity and hazards that CeONPs and other nanoparticles poses to human health and the environment. Exposing pure CeONPs to a human bronchus epithelial in vitro model (BEAS-2B cell line) induced cell death with decreased intercellular glutathione (GSH) reported [73]. This enzyme is an effective ROS scavenger and playing a key role in the inflammatory and oxidative stress pathways [73]. Another study found that CeONPs induced oxidative damage and led to decreased lifespan in an in vivo model (*Caenorhabdotis Elegans* cell line) [74]. Similarly, exposure of CeONPs decreased cell viability and increased ROS production in a human skin melanoma in vitro model (*A375* cell line) [75]. In acidic conditions the antioxidant ability of CeONPs is lost, behaving instead much like a strong oxidant, which may influence the oxidation of intracellular and extracellular components to induce apoptosis [76]. This ability to become cytotoxic has been found to induce oxidative stress, could be attributed to cancer cells being more acidic than normal cells [70,77]. The bifunctional characteristic of CeONPs allows it to exhibit both ROS scavenging and cytotoxic effects is possible due to its ability to change structural composition in valency depending on the environment [78,79]. These studies provide insight into the various therapeutic applications of CeONPs, which is further examined in the final period of CeO_2_ research.

#### 2.4.4. Time Period 2014–2020—“*Cerium Oxide* and Pathology”

This period yielded the highest number of publications (*n* = 69), with the highest number of publications (*n* = 22) from a single year (2020). Following on from the previous period, the broad themes are the same, with most publications highlighting the environmental/industrial applications of CeONPs, with several studies focusing on its biomedical uses.

Environmental/industrial applications of CeO_2_ make up most of the publications in this period (*n* = 63). Like the previous period, CeONPs were investigated as renewable resources, efficient fuel cells and remediating the environment through implementing hybrid CeONPs complexes. Of particular interest is the advancement of the single atom catalyst, with one study using platinum single atoms on the surface of CeONPs (surface modified/functionalized support) demonstrating higher reactivity, better selectivity and less agglomeration when heated, resulting in a superior compound compared to the platinum/CeONPs nanocomposite complex [80]. The fluorite lattice structure of CeONPs were exploited in this period with studies using various additives (e.g., titania/CeONPs, lanthanum/CeONPs nanocomposites and nickel, zirconia oxide/CeONP nanocomposites surface functionalized with palladium) for methane combustion and removal of organic dye (e.g., Rhodamine-B dye) with enhanced catalytic performance compared to bare CeONPs [81,82,83]. Another study found that CeONPs supported platinum-selenium clusters (as surface modified/functionalized additives) extending the catalytic reaction which unearthed full regeneration, increased dispersibility and stability of the complex for CO oxidation [84]. The use of the core–shell nickel encapsulated between silica oxide/CeONP nanocomposites was useful in reforming biogas, leading to increased nickel dispersion and reductivity compared to nickel-silica or nickel-ceria alone, therefore resulting in the development of a potential sustainable hydrogen fuel source [85]. All these studies extend from the previous periods of CeONPs research further highlighting the versatility of CeONPs, leading to additional advancements in catalytic control and improved performance from an environmentally conscious and industrial perspective.

The remaining publications highlight the biomedical applications of CeONPs, engineering the nanoparticles to enhance their biological activities, as well as, continuingly, the engineering of CeONPs for environmental/industrial applications. Following on from the previous period, montmorillonite loaded CeONP nanocomposites and porphyrin surface modified/functionalized CeONPs have been explored as diagnostic tools to detect hydrogen peroxide (H_2_O_2_) and glucose [86,87]. These nanostructures were found to exhibit peroxidase (enzyme that reacts with H_2_O_2_ to catalyse oxidation of a number of inorganic compounds in samples) like catalytic activity demonstrating a colorimetric sensitive and specific method, indicating its potential for biochemical assays, clinical diagnosis and environmental monitoring [86,87]. Advancing the idea of CeONP as a therapy in cancer in this time period, one study demonstrated the use of manganese doped-CeONP nanocomposites inducing higher cytotoxicity effects in an adenocarcinoma in vitro model (MCF7 cell line) compared to bare CeONPs [88]. The enhanced cytotoxic effect is likely due to the higher oxygen vacancies generated from adding manganese, producing increased ROS generation which target and kill the cancerous cells, therefore demonstrating its potential as a promising cancer targeting therapy [88]. The bifunctional properties of CeONPs allow it to change its role from antioxidant to pro-oxidant, depending on its surrounding environment, with an acidic environment inducing a pro-oxidant cytotoxic property allowing it to target cancer cells [89]. CeONPs as a drug delivery agent was also investigated for its use in acute kidney injury (AKI) [90]. In this instance, exposure of surface modified/functionalized CeONPs with triphenylphosphine, conjugated with an ROS-responsive organic polymer (PEG—polylactic acid-glycolic acid copolymer), and further loaded with atorvastatin, resulted in protection against tubular cell apoptosis and necrosis in an LPS-induced AKI in vivo model. This study showed the successful modification of CeONPs to target the kidney and selectively release the drug in response to the presence of ROS [90]. Another study highlighted CeONPs as an effective drug delivery agent, using CeONPs encapsulated within zeolithic imidazole framework-8. This nanostructure could penetrate the blood–brain barrier (BBB), accumulate in the brain tissue and reduce oxidative damage and apoptosis of neurons, in an ischemic stroke middle cerebral artery occlusion (MCAO) in vivo mouse model [91]. This study confirms those of Kim and collegues in the previous time period of CeONP potential as a therapy for ischemic stroke. CeO_2_ as neuroprotective agent has further been investigated, with one study adding triphenyl phosphonium (TPP)-conjugated CeONPs as potent ROS/RNS scavengers due to its recyclable ability, reducing oxidative stress in an in vivo Alzheimer’s disease (AD) mouse model (5XFAD transgenic mice) [92].

The advancements in nanoparticle engineering in this period is clear, with extensive use of doping, nanocomposite, surface functionalized or hybrid (doped and nanocomposite) nanostructures, designed for their specific purpose, considering the morphology, surface texture and additives. The biomedical applications of CeONPs in this period, for their ROS scavenging abilities, as anti-inflammatory therapy, as a diagnostic testing method and of particular interest, the neuroprotective properties for neurological diseases and neurodegeneration.

#### 2.4.5. Author Network and Countries with Most Citations for Cerium Oxide Research

**Figure 5:**
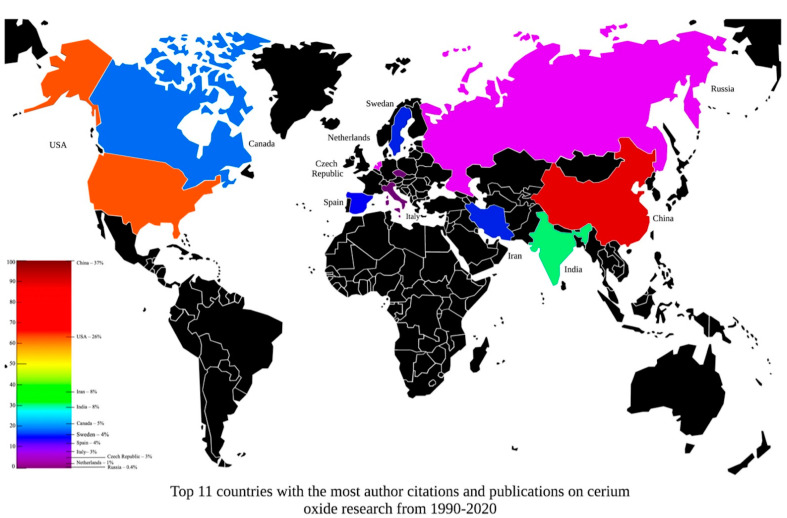
Top 11 countries, with the highest citations (minimum 10) and documents (minimum 10) per authors, grouped by country affiliations. Extrapolated from the dataset obtained from the WoS database, using the term “cerium oxide or ceria or nanoceria or nano ceria” in the years 1990–2020. Accessed on the 9 October 2020.

The systematic literature search found a wide range of ground-breaking research into CeONPs over the last 30 years identifying advancements in nanoparticle engineering which has increased the understanding of CeO_2_ properties whilst uncovering a larger body of scientific work for renewable resources, solid fuel cells, along with its potential as cancer, inflammatory and neuroprotective therapies. To evaluate the authors contributing to CeO_2_ research, an author network was created from the dataset obtained from the WoS database (7862 publications) using the term “cerium oxide OR ceria OR nanoceria OR nano ceria” in the years 1990–2020 (accessed on the 17 September 2020). The data from this search was imported into the VOSviewer software, where a bibliographic database search was completed for co-authorship using full counting method resulting in a total of 25,580 authors. The authors were further refined by a minimum of 10 documents and 10 citations per author (using as an inclusion criteria for peer-acknowledged quality, h-index) which resulted in 68 authors [93]. The author’s affiliations and number of citations were recorded and the percentage of citations per country was calculated and ordered by most to least citations (Figure 5). The top 11 countries were identified, showing the highest number of citations from China, encompassing a total of 37%, indicating that China has led the world in CeO_2_ research over the last 30 years. Other countries like USA (26%), Iran and India (8%), Canada (5%), Sweden and Spain (4%), Italy (3%), Czech Republic (3%), Netherlands (1%) and Russia (0.4%) have also contributed to CeO_2_ research over the past 30 years.

## 3. Discussion

**Figure 6:**
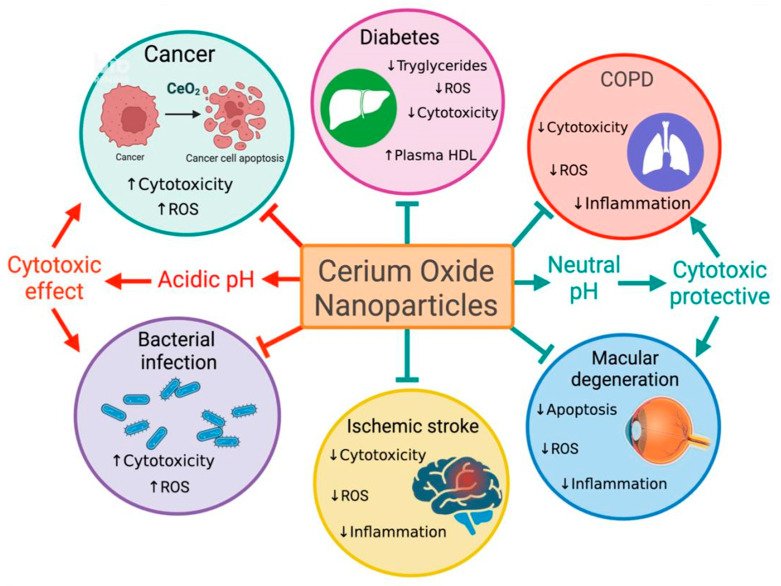
Graphical depiction of the various biomedical applications of cerium oxide nanoparticles as therapies for a wide range of pathologies including cancer, diabetes, cardiovascular pulmonary disease, macular degeneration, ischemic stroke and bacterial infection due to the bifunctional nature of CeO_2_. In acidic conditions the antioxidant ability of CeONPs is lost, behaving instead much like a strong oxidant, which may influence the oxidation of intracellular and extracellular components to induce apoptosis. This ability to become cytotoxic has been found to induce oxidative stress, could be attributed to cancer cells being more acidic than normal cells.

This systematic literature analysis found that the research on “*cerium oxide*” has developed over the past three decades from initially focusing on understanding the unique physicochemical properties of cerium oxide, into the extensive environmental and industrial applications, and finally focusing on CeONPs as a biomedicine. The biomedical applications of CeONPs in the more recent time periods have uncovered the advancements in nanoparticle engineering (e.g., dopants, coating, surface functionalization, core–shell structures, nanocomposites, and hybrid structures). CeONPs additives were able to increase the already remarkable biological effects of CeONPs as cancer therapeutics, for therapy in inflammatory disease and as diagnostic tools. Moreover, its unique ability to induce cytotoxic effects to cancerous cells whilst inducing protective effects against cytotoxicity in non-cancerous cells indicates its potential for development as therapy for range of pathologies (Figure 6).

The scientific literature on CeO_2_ over the past 30 years, presented in the topic modelling found the catalytic applications of CeONPs were prominent in the WGS reaction. However, most of the topics that were identified referred to the potential biomedical applications, highlighting its oxidative, antioxidant, catalytic and anti-cancer properties. Further analysis of the topic models using the VOSviewer software, identified nine clusters, highlighting the various fields and developments of CeONP applications largely focusing on the surface and morphological alterations of the nanostructures across various environmental/industrial and biomedical fields.

The SciMAT software was used for a systematic literature analysis, established themes for each time-period. The first period (1990–1997) focused on the environmental/industrial applications of doped and undoped CeONPs, for the enhancement of solid fuel electrolyte cells found to increase the efficiency of the WGS reaction to replace non-renewable resources (e.g., coal, natural gas, oil and nuclear energy) [94]. Replacing non-renewable resources is a major problem for humanity, however, is necessary to create an environmentally sustainable lifestyle. The WGS reaction is significant for this, reforming hydrocarbons to produce hydrogen as an energy source [94]. Fuels produced from hydrogen can be used as direct replacements for oil and gas as low carbon emitting alternatives, which is more environmentally sustainable [95]. CeONPs have been found to be a more effective catalysts for the WGS reaction compared to noble gases (e.g., platinum and manganese) and transition metals (e.g., copper) due to its oxygen storage capacity, facilitated by the ability of transitioning from the trivalent (Ce^3+^) to the tetravalent (Ce^4+^) state [96]. This property is enhanced by doping CeONPs with various metal (e.g., platinum, manganese, nickel, cobalt, zirconia and gold) and non-metal (e.g., silica and selenium) catalysts, achieving a powerful catalytic system with increased surface area exposing more oxygen vacancies, resulting in long-term stability and reproducibility, as well as higher CO conversion activity at lower temperatures [94,97,98,99]. In light of these enhanced properties, doping, another manufactured additive is highly utilized in this period and has been shown to be crucial in influencing and controlling the surface reactivity of CeONPs. The doping of CeONPs in this period, focused on increasing the oxygen vacancies (active sites) overall improving catalytic performance and compatibility. This suggests that increasing oxygen vacancies for improved performance is an alteration that is still investigated currently with various studies using additives like titania, lanthanum, zirconia, palladium, yttrium and zinc CeONP structures which have increased oxygen vacancies as environmental remediators (e.g., degrade crystal violet dye, Rhodamine-B dye and methane combustion) [81,82,83,100].

The environmental/industrial and biomedical application using CeONPs in the second period (1998 to 2005) continued to focus on the manufacturing alterations of CeONPs to further enhance its performance. The environmental/industrial applications of CeONPs highlight the influence that chemical composition has on its catalytic activity regarding the WGS reaction and environmental remediation. As the catalytic performance relies heavily on oxygen vacancies, the crystal plane and nanorod morphologies of CeONPs were found to exhibit the highest catalytic performance compared to crystal lattice, nanocube and nanosphere structures [36,37,38]. The increased oxygen vacancies had significant impact on the electrostatic surface charge of the nanostructures which affect the agglomeration rate and overall stability of the complex [96]. Nanocomposites incorporate nanosized particles into a matrix, which has increased oxygen vacancies compared to single nanoparticle structures, but also has enhanced reducibility due to the interactions that occur between the nanoparticles in the matrix further improving catalytic performance [18]. CeONP nanocomposites combined with copper, nickel, zirconia, aluminium, platinum and gold nanoparticles have been extensively used for the WGS reaction in this period [101]. CeONPs nanocomposites are still investigated currently in renewable energies, with a study highlighting the use of silica oxide/CeONPs nanocomposites in reforming biogas, and a potential sustainable hydrogen fuel source [85].

Consistent with the environmental/industrial applications of CeONPs in this period (1998 to 2005), manipulations of CeONPs size, morphology, and additives were investigated for its biomedical application, particularly as a cancer targeting therapy. CeONPs have been found to have anti-tumour properties, becoming cytotoxic towards cancer cells pro-oxidant producing ROS to target them (i.e., pro-oxidant), whilst having little to no effect to the surrounding healthy cells [89]. This is due to the enzymatic abilities in effectively switching valence states from the Ce^3+^ (reduced form) to the Ce^4+^ (oxidised form) donating an electron [79,102]. Nanoparticle studies have found that sharp edged, large nanostructures are less biocompatible, compared to smooth surface smaller nanostructures, as they can inflict mechanical damage on cell membranes and organelles while triggering an immune response [96]. A study using ultrafine bare CeONPs found that the nanoparticles induced cytotoxicity on a breast carcinoma in vitro model (MCF-7 cell line) [40]. Another study showed that tumour cells create an acidic environment, which induces oxidant like behaviour in CeONPs. This study used murine fibrosarcoma tumour cells (WEH164 cell line) which were injected in the flank of a murine in vivo model (BALB/c). The CeONPs (<50 nm) were extremely efficient in targeting and aggregating at tumour site, finding that enhanced permeability and retention plays a crucial role in delivering the CeONPs to tumour cells [103]. These studies provide further evidence for CeONPs as potent ROS scavengers and potential as effective cancer therapies. Of note, doping CeONPs for biomedical applications surfaced in this period, showing clear advantages, in preventing agglomeration and toxicity compared to undoped equivalents [96]. This is consistent with a study that used dextran-coated CeONPs in an osteosarcoma in vitro model (MG-63 cell line), finding that these nanostructures induced increased toxicity compared to bare-CeONPs. This effect was shown to be dose dependent with increasing toxicity as dextran coating concentration increased [104]. Another study using polymer-doped CeONPs in a human lung fibroblasts in vitro model (ATCC and mRC-9 cell lines) demonstrated rapid adsorption, increased retention time and increase dispersion, compared to the bare CeONP equivalent [39]. Doping CeONPs has been found to improve its antioxidant abilities, more effectively scavenging ROS/RNS, enhancing solubility, stability and dispersion of the nanoparticles, which is further investigated in successive time periods [105]. These studies suggest that CeONPs are effective anti-cancer therapies due to their unique ability to differentiate between healthy and cancerous cells with polymer coated CeONPs showing enhanced efficacy in this ability to target the cancerous cells.

The third period (2006 to 2013), saw further developments in the environmental/industrial applications of CeONPs as a heterogenous catalyst (multiple catalysts in a nanocomposite structure). The use of metal oxide/CeONPs nanocomposites as heterogenous catalysts were used having increased oxygen storage for the WGS reaction [106]. This is seen in a study using metal (platinum, titania, gold) oxide/CeONPs nanocomposites to generate a more efficient catalyst, as the hybrid materials incorporated into the crystal lattice structure provide active support and new oxygen vacancies at the metal oxide/CeONPs interface [106,107]. The biomedical applications in this period, provided support for CeONPs as an anti-cancer agent, however, transitioned into targeting inflammatory driven diseases and wound healing, highlighting its antioxidant abilities. CeONPs are effective cancer targeting therapies due to the pro-oxidant properties which they exhibit [70,77]. These mechanisms of CeONPs are highly reliant on the pH of the surrounding environment being more acidic (pH 6) which is attributed to cancer cells being more acidic than normal cells [108,109,110]. In a more alkaline pH (e.g., pH 7–9) the antioxidant abilities of CeONPs are exhibited, inducing strong ROS scavenging properties [76]. This bifunctional property is due to alternating valence states and hence the ability to regenerate oxygen vacancies, which are active sites for redox reactions (ROS/RNS scavenging) to take place [5,96]. This property of CeONPs is important because oxidative stress is considered central to the progression of chronic inflammation and inflammatory diseases [111]. A study used micro RNA 146a (miR146a)/CeONPs nanocomposites to treat colitis on in vivo murine model (C57BL/6), finding that the nanostructure exhibited an anti-inflammatory and antioxidant activities, decreasing inflammatory cytokines; tumour necrosis factor (TNF) and interleukin 6 (IL-6), coinciding with the reduced oxidative stress [112]. Similarly, in vitro models, adipocyte (3T3-L1 cell line) and myoblast (C2C12 cell line) of diabetes mellitus were exposed to selenium/CeONPs nanocomposites, showing a decrease in extracellular ROS production [113]. These studies show that CeONPs nanocomposites can alleviate oxidative stress and inflammation responses. Oxidative stress has been associated with increased bone loss, as well as limiting bone repair, being correlated to low-grade chronic inflammation. Spherical CeONPs exhibited protective effects, by enhancing cell proliferation as well as osteogenic differentiation and mineralization on a human bone-derived mesenchymal stem cell model (hBMSCs cell line). This study showed the positive effects of CeONPs for tissue and bone regeneration under acute or chronic conditions [114]. Taken together, these studies demonstrate the important biological activities of CeONPs as anti-inflammatory and antioxidant agent, demonstrating the nanoparticles potential use in a wide spectrum of inflammatory-associated diseases. The development of CeONPs nanocomposites in this period highlights advancements in nanoparticle engineering.

The most recent period (2014–2020) showed a significant development in environmental/industrial applications of CeONPs in the single atom catalysts for the WGS reaction. Single atom catalysts, have been developed to increase catalytic performance, due to the increased dispersion rate and the active sites encapsulated on the support structure, achieving higher turnover rates compared to larger nanoparticles [94]. One study used a platinum single atom, supported on CeONP catalysts (1.7 nm in diameter), which reported a high number of surface oxygen vacancies. This structure exhibited increased dispersibility, due to the synergistic effects of platinum and CeO_2_ [115]. Noble metals atomically dispersed on solid oxide supports, have become the superior form of heterogenous catalysis. An example is the palladium doped CeONPs single atom catalysts, which have been found to have increased interactions between the metal-support, a crucial property for maintaining stability [116].

The CeONPs biomedical applications in this period are the most interesting, identifying a clear trajectory of research into CeONPs as therapies for neurological diseases and in particular neurodegenerative diseases. CeONPs have emerged as potential neurodegenerative therapies due to the ease at which these nanoparticles can be manipulated to adjust the size and additives, for passage through the BBB. One study investigated the effects of CeONPs in an anaplastic astrocytoma in vitro model (grade II glioma), displaying selective cytotoxicity targeting astrocytoma cells [117]. PEG coated CeONPs can easily cross the BBB, therefore various studies exploring treatment for ischemic stroke and multiple sclerosis (MS) have emerged [118]. Studies tested these nanoparticles in in vivo murine models of ischemic stroke and MS effectively scavenging ROS/RNS and reducing overall oxidative stress injury [117,118]. Another study used citrate EDTA stabilized CeONPs in an in vitro murine model (*SOD1G93A* cell line) of amyotrophic lateral sclerosis (ALS), finding that the ROS/RNS scavenging ability assisted in prolonging lifespan after muscle weakness was observed [118]. These studies demonstrate that CeONPs are able to induce both protective and cytotoxic effects efficient against oxidative stress/inflammatory driven diseases as well as cancer [75]. This dual effect of CeONPs to be both protective and selectively cytotoxic is remarkable and is found to rely on the surrounding pH [89]. In acidic conditions the cytotoxic ability of CeONPs is activated whilst the antioxidant ability is lost, behaving instead much like a strong oxidant which influences the oxidation of intracellular and extracellular components to induce apoptosis in the presence of cancerous cells [76]. This ability to become cytotoxic has been found to induce oxidative stress, could be attributed to cancer cells being more acidic than normal cells [70,77]. The bifunctional characteristic of CeONPs allows it to exhibit both ROS scavenging and cytotoxic effects, due to its ability to change structural composition in valency and its interaction with the surrounding environment [78,79,119]. These properties makes it an extremely versatile compound for various biomedical applications, including an efficient drug targeting device, and in oxidative stress diseases like neurodegeneration [79].

CeONPs as a therapy for neurodegeneration and particularly AD has emerged in recent years due to the driving pathological features where oxidative stress and inflammation is now recognized as playing a key role in disease progression [120,121]. Various studies have tested CeONPs in human neuroblastoma in vitro model (*SH-SY5Y* cell line) treated with amyloid-beta (Aβ) protein (protein that aggregates and forms Aβ senile plaques, a hallmark pathology of AD) [118,122]. Similarly, the use of CeONPs but coated with an anti-amyloidogenic agent, inhibited amyloidogenic activity with no toxicity present in an in vitro human glial model (*U87MG* cell line) [123]. These studies highlight CeONPs antioxidant, anti-inflammatory and neuroprotective properties, as well as the ability to permeate the BBB, for its development as a promising therapy for neurological and/or neurodegenerative diseases like AD.

**Figure 7:**
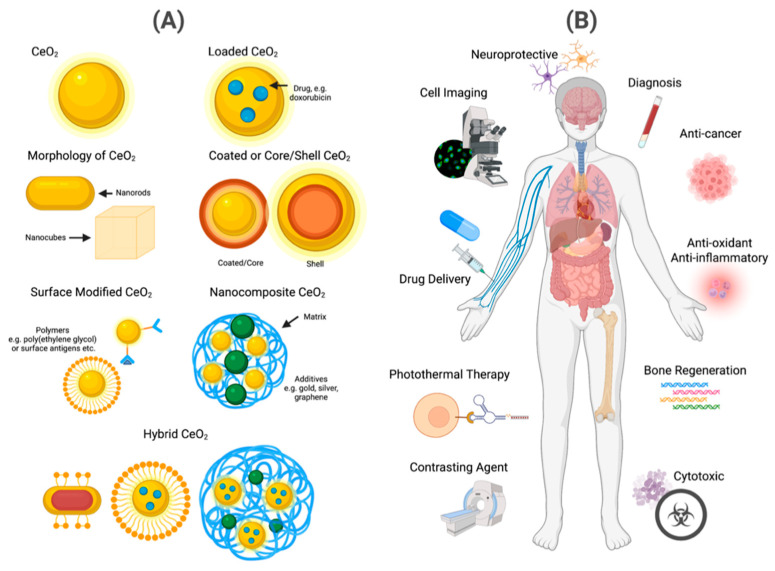
(**A**) Graphical representation of the various nanoparticle engineering manipula-tions of cerium oxide that have been implemented in (**B**) various biomedical applications (e.g., drug delivery agent, diagnosis, anti-cancer agent and contrasting agent).4. Materials and Methods.

The advancements in nanotechnology over the years have led to the extensive applications of CeONPs in the environmental/industrial and biomedical fields. Whilst the use of CeONPs have mostly been beneficial, adverse effects have been reported. For example, increased dosage and sized of CeONPs was associated with varied toxicity levels and apoptosis in human neuroblastoma cells (*IMR32* cell line) [124]. Similarly, oxidative stress and cytotoxicity was increased following exposure to CeONPs in human lung adenocarcinoma cells (*A549* cell line) [125]. In contrast, CeONPs showed neuro- and cardio-protective effects through suppression of decrease ROS and oxidative stress [71,126]. These studies indicate that whilst CeONPs has great potential for biomedical applications, future research is still necessary to optimize CeONPs as a therapy, particularly focusing on dosage, administration, particle composition, size and shape which have known effects on their interactions with molecular mechanisms of various cells and tissues in biological systems (Figure 7).

## 4. Materials and Method

### 4.1. Topic Modelling

The PubMed (https://pubmed.ncbi.nlm.nih.gov/ (accessed on 17 September 2020)) database was used to search the term “cerium oxide OR ceria OR nanoceria OR nano ceria” from 1990 to 2020. The publications extracted (129 publications; included research and review articles) were exported as a PMID list file and imported into the SWIFT (Sciome Workbench for Interactive Computer-Facilitated Text-mining)—Review software (https://www.sciome.com/swift-review/), where the articles were segregated based on keywords, then organized into topic models of cerium oxide research and ranked in order from most to least prevalent topics. To enlarge the search range, other databases were used as a source of bibliographic data. All databases were accessed in September and October 2020.

### 4.2. Bibliometric Analysis

Bibliographic data (title, abstract, all citations) for the period of 1990–2020 were exported (txt file) from the Web of Science Core Collection (WoS) database (accessed on the 17 September 2020) using the search “cerium oxide or ceria or nanoceria or nano ceria”. The dataset was then imported into the VOSviewer software (www.vosviewer.com). Any duplicates in the search were removed and a bibliometric analysis (yielding 7862 publications), was performed based on co-occurrence of authors keywords in the paper title (9253 keywords) using full counting, which was then further refined to a minimum of 20 occurrences, yielding 144 keywords.

### 4.3. Longitudinal Study

**Figure 8:**
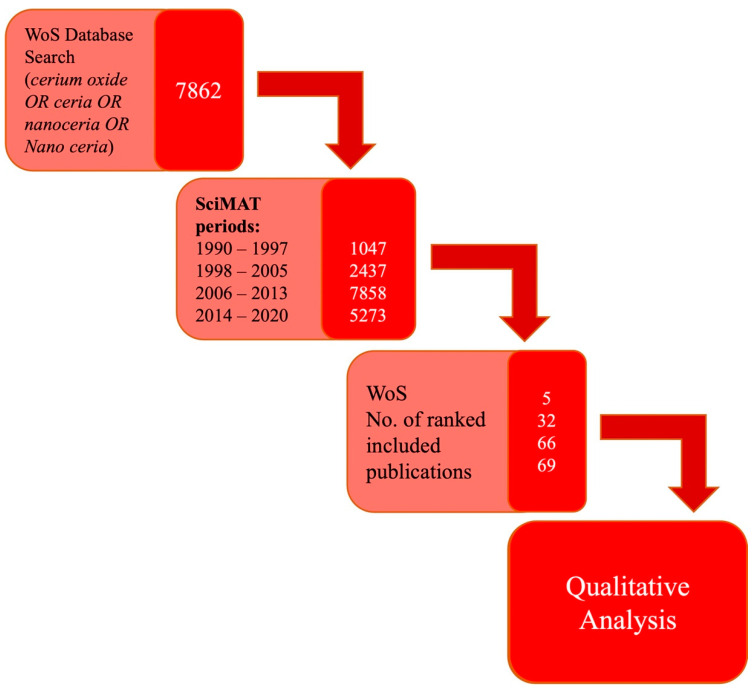
Graphical depiction of the systemic literature review process of the WoS database (numbers represent the number of publications analysed at that step).

A bibliographic search was performed to undertake a review of the literature over the past three decades, using the Scopus database (accessed on the 9 October 2020) with the search term “cerium oxide OR ceria OR nanoceria OR nano ceria” from 1990 to 2020. The dataset was exported (authors keywords) and imported into Science Mapping Analysis Software Tool (SciMAT) (https://sci2s.ugr.es/scimat/) software for analysis. Within the data set, identical and similar words were grouped to identify the literature trends and themes. Publications were reviewed in four time periods (1990–1997, 1998–2005, 2006–2013 and 2014–2020) for ease of investigation in a chronological manner, using the workflow presented in Figure 8. Normalization of all the publications was completed using the analysis function, focusing on keywords and specifically authors keywords, with a frequency reduction minimum of 10, a co-occurrence matrix, the edge value reduction of 8, normalization of association strength, simple algorithm centres and core mapping was used. The quality index (h-index) was used, and for the longitudinal analysis, the inclusion index was used on the overlapping map and the Jaccard’s index was used for the evolution map. The important motor-themes (main themes) in each period were identified by their location in the upper right-hand quadrant of the strategic diagram generated by SciMAT software.

### 4.4. Systematic Review of Literature

Using the four main motor themes generated from the SciMAT software, a systematic review of the peer-reviewed literature was carried out using the Scopus database (accessed on the 9 October 2020). In this search, the four main motor themes identified in SciMAT were included in the time frame of 1990–2020 (e.g., “cerium oxide OR ceria OR nanoceria OR nano ceria AND doping additives AND catalyst AND reactive oxygen species AND pathology, 1990–2020”). The publications were restricted to the relevant time period related to the motor theme. The publications were assessed according to the Field Citation Ratio (FCR) for all time periods. A total of 172 publications were included in this analysis. A FCR value of greater than 6 was included, indicating peer-acknowledged quality for each publication in this review. Reviews, editorials and short communication were excluded from further analysis.

### 4.5. Author Network

To establish a co-authorship network, the dataset obtained from the WoS database (new format, RIS file), from the search “cerium oxide OR ceria OR nanoceria OR nano ceria” in the years 1990–2020 (accessed on the 9 October 2020) was imported into VOSviewer software. A bibliographic database search, using full counting method, was then completed for co-authorship which resulted in 25,580 authors identified. The authors were further refined by a minimum of 10 documents and 10 citations per author (used as the inclusion criteria for pear-acknowledge quality, h-index) yielding 68 authors. The authors affiliations, number of citations, along with the affiliated countries were recorded and the percentage of citations per country were calculated to determine the frequency of “magnetite’ research around the world. The percentage of citations per country was calculated and ordered by most to least.

## 5. Conclusions

The research over the last three decades on CeONPs have made progress in environmental and industrial applications as an effective catalyst, for environmental remediation and replacing fossil fuels with renewable resources. The biomedical applications have also increased our understanding of the properties and characteristics of CeONPs, leading to the manipulation and enhancements of CeO_2_ nanostructures. The unique redox property of CeO_2_ makes it a potent antioxidant when compared with other ROS modulators, including promising applications in cancer, inflammatory diseases, neurological diseases, and neurodegeneration. The key to CeONPs as a neurological therapy, is its facilitation to cross the BBB, which is easily obtainable due to the advancements in nanotechnological engineering. Further research in both in vitro and in vivo studies need to be undertaken, to fully understand the exact anti-inflammatory mechanism that CeO_2_ possesses and the synergistic therapeutic effect of oxidative stress reduction. This will aid in developing therapies for various diseases, showing remarkable potential as a biomedicine for inflammatory diseases, but also for neurodegenerative diseases like AD.

## Figures and Tables

**Table 1:** Summary of the number of papers identified in searchers of different databases (PubMed, WoS and Scopus) using the search terms “cerium oxide OR ceria OR nanoceria OR nano ceria” from the years 1990–2020.

Search Terms	PubMed	WoS	Scopus
*Cerium oxide*	122	27,674	22,467
*Cerium oxide* and nanoparticles	59	16,252	738
*Cerium oxide* and pathology	17	13,350	193
*Cerium oxide* and toxicity	29	14,533	624

Notes: Accessed on the 17 December 2020, and covered the article, title, abstract, and keywords.

**Table 2:** Top 19 topic models generated from PubMed dataset (129 publications) by SWIFT-Review software using the search terms “cerium oxide OR ceria OR nanoceria OR nano ceria”. This search was refined to review, clinical trials, meta-analysis, and research articles. The topics have been ordered by number of publications contributing to the topic model in descending order, with topic words and themes established.

Topic No.	Topic Word	No. of Publications in Topic Model	Brief Description of Topic
34	Applications, properties, synthesis, review, biomedical, advances	101	Properties for biomedical applications
97	Nanoparticles, oxide, cerium, review, activity, properties, oxidative, research	85	Oxidative Properties
8	Ceria, materials, surface, application, energy, properties, systems, structure, material	57	Surface materials and structure
84	Current literature, include, increase, therapies, existing conditions	49	Therapies
5	Antioxidant, potential, species, effects vivo reactive, stress, biological, ROS, oxygen	46	Antioxidant properties
1	Therapy, clinical outcomes, patients, improved, scientific approaches	38	Clinical approaches
93	Catalysts, catalytic, reactions, oxidation, reaction, ceria-based, catalyst, high activity, organic	28	Catalytic properties
18	Medicine, role, toxicity, regenerative, promising, industry, recent	26	Medicinal therapies
21	HIV, trial, study, incarcerated, months, release, randomized, results, observed treatment	23	Randomized trials
51	Challenges, cells, order, progress, systems, target, addition, improve, medical barriers	23	Targeted treatments
28	Model, studies, hydrogen, techniques, design, kinetics, water-gas, knowledge, modelling, advanced	21	Kinetic studies
70	CEONPs, surface, oxygen, vacancies, lattice, results, experimental, presence, evidence	20	Surface structure
86	Nanoceria, cells, CEONPs, particles, anti-cancer, data, point, improve, agent normal	19	Anti-cancer agent
48	Design, role, aims, emissions, reviews, interest, carbon, focusing, develop, materials	18	Carbon emission materials
69	Health, effects, exposure, fuel, engineered, risk, toxicological, studies, air	18	Air pollution
72	Synthesis, methods, synthetic, number, template, products, implications, function, metals, enhanced	18	Synthesis methods
38	Treatment, development, growth, pathway, inhibitors, target, tumour, kinase, receptor, phase	17	Tumour kinase pathway
63	Review, systematic, studies, outcomes, articles, results, reported, included published, gaps	17	Systematic reviews
85	Control, research, populations, article, needed, major, practice, describes, provided, discussion, gaps	16	Research gaps

Notes: Accessed on the 17 September 2020. Refined to review, clinical trials, meta-analysis, and research articles.

**Table 3:** Summary of the word clusters identified using VOSviewer and WoS dataset using the search term “cerium oxide OR ceria OR nanoceria OR nano ceria”. The network analysis from 7862 publications from 1990 to 2020. The clusters are also represented in a visualisation map (Figure 2).

Cluster	Lay/Description	Keywords
1	Biocompatibility as a biomedical application	Antioxidant, apoptosis, biocompatibility, blood-brain-barrier, catalase, cerium oxide nanoparticles, cytotoxicity, drug delivery, genotoxicity, inflammation, nanoceria, nanomedicine, nanotechnology, oxidative stress, translocation
2	Surface coating additives	Catalyst, cerium oxide, cobalt oxide, copper, hydrogen production, hydrogen peroxide, hydrogen production, interface, methane, nickel, oxygen reduction reaction
3	Catalytic properties remediation	Biodiesel, catalytic oxidation, cathode, ceria nanoparticles, conductivity, electrolyte, manganese oxide, metal oxides, methanol, palladium, reaction mechanism, stability
4	Manufacturing alterations	Catalysis, ceramics, coatings, copper oxide, crystal structure, defects, doping, nanostructures, optical properties, oxides, photocatalytic activity, ramen spectroscopy, solid solution, structure
5	Advancements in thermal stability	Adsorption, antibacterial activity, co-precipitation, coating, corrosion, resistance, electron microscopy, hydrothermal synthesis, kinetics, mechanical properties, metal oxide, microstructure, thermal stability, X-ray diffraction
6	Advancements in manufacturing (nanocomposites) improved efficiency	Corrosion, electrochemical sensors, electrodeposition, ionic conductivity, lanthanum oxide, mechanism, nanocomposite, oxygen vacancies, photocatalysis, rare earth metals, reduced graphene oxide, thin film, titanium dioxide, zinc oxide
7	Degradation, air pollutant and biomedical applicant	Catalytic ozonation, cerium dioxide, degradation, graphene oxide, inhalation, nanomaterial, nanozyme, wound healing
8	Catalytic interaction of doped cerium oxide	Doped ceria, heterogenous catalysis, metal-support interaction, oxygen vacancy, sintering, synergistic effect
9	Antioxidant activity	Aggregation, anti-bacterial, antioxidant activity, morphology, ROS, silver nanoparticles, toxicity

Note: Accessed on the 17 September 2020.

## Data Availability

Not applicable.

## References

[1] Dahle J.T., Arai Y. (2015). Environmental geochemistry of cerium: Applications and toxicology of cerium oxide nanoparticles. Int. J. Environ. Res. Public Health.

[2] Karakoti A.S., Kuchibhatla S.V.N.T., Babu K.S., Seal S. (2007). Direct Synthesis of Nanoceria in Aqueous Polyhydroxyl Solutions. J. Phys. Chem. C.

[3] Murray E.P., Tsai T., Barnett S.A. (1999). A direct-methane fuel cell with a ceria-based anode. Nature.

[4] Xu C., Qu X. (2014). Cerium oxide nanoparticle: A remarkably versatile rare earth nanomaterial for biological applications. NPG Asia Mater..

[5] Dhall A., Self W. (2018). Cerium Oxide Nanoparticles: A Brief Review of Their Synthesis Methods and Biomedical Applications. Antioxidants.

[6] Pourkhalili N., Hosseini A., Nili-Ahmadabadi A., Hassani S., Pakzad M., Baeeri M., Mohammadirad A., Abdollahi M. (2011). Biochemical and cellular evidence of the benefit of a combination of cerium oxide nanoparticles and selenium to diabetic rats. World J. Diabetes.

[7] Pešić M., Podolski-Renić A., Stojković S., Matović B., Zmejkoski D., Kojić V., Bogdanović G., Pavićević A., Mojović M., Savić A. (2015). Anti-cancer effects of cerium oxide nanoparticles and its intracellular redox activity. Chem. Biol. Interact..

[8] Nelson B.C., Johnson M.E., Walker M.L., Riley K.R., Sims C.M. (2016). Antioxidant Cerium Oxide Nanoparticles in Biology and Medicine. Antioxidants.

[9] Han J., Kang H.-J., Kim M., Kwon G.H. (2020). Mapping the intellectual structure of research on surgery with mixed reality: Bibliometric network analysis (2000–2019). J. Biomed. Inform..

[10] Shen Z., Wu H., Chen Z., Hu J., Pan J., Kong J., Lin T. (2022). The Global Research of Artificial Intelligence on Prostate Cancer: A 22-Year Bibliometric Analysis. Front. Oncol..

[11] Nugraha A.S. (2021). Bibliometric Analysis of Magnetite Nanoparticle Production Research During 2017–2021 Using Vosviewer. Indones. J. Multidiciplinary Res..

[12] Li Z., Hu M., Song H., Lin D., Wang Y. (2021). Toxic effects of nano-TiO(2) in bivalves-A synthesis of meta-analysis and bibliometric analysis. J. Environ. Sci..

[13] Eguchi K., Setoguchi T., Inoue T., Arai H. (1992). Electrical properties of ceria-based oxides and their application to solid oxide fuel cells. Solid State Ion..

[14] Trovarelli A. (1996). Catalytic Properties of Ceria and CeO_2_-Containing Materials. Catal. Rev..

[15] Sayle T.X.T., Parker S.C., Catlow C.R.A. (1994). The role of oxygen vacancies on ceria surfaces in the oxidation of carbon monoxide. Surf. Sci..

[16] Yashima M., Arashi H., Kakihana M., Yoshimura M. (1994). Raman Scattering Study of Cubic–Tetragonal Phase Transition in Zr1−xCexO2 Solid Solution. J. Am. Ceram. Soc..

[17] Conesa J. (1995). Computer modeling of surfaces and defects on cerium dioxide. Surf. Sci..

[18] Malhotra B.D., Ali M.A., Malhotra B.D., Ali M.A. (2018). Chapter 5—Nanocomposite Materials: Biomolecular Devices. Nanomaterials for Biosensors.

[19] El Morabet R., Nriagu J. (2019). Effects of Outdoor Air Pollution on Human Health. Encyclopedia of Environmental Health.

[20] Corma A., Atienzar P., García H., Chane-Ching J.Y. (2004). Hierarchically mesostructured doped CeO2 with potential for solar-cell use. Nat. Mater..

[21] Kharton V.V., Figueiredo F.M., Navarro L., Naumovich E.N., Kovalevsky A.V., Yaremchenko A.A., Viskup A.P., Carneiro A., Marques F.M.B., Frade J.R. (2001). Ceria-based materials for solid oxide fuel cells. J. Mater. Sci..

[22] Steele B.C.H. (2000). Appraisal of Ce1−yGdyO_2_−y/2 electrolytes for IT-SOFC operation at 500 °C. Solid State Ion..

[23] Li Y., Fu Q., Flytzani-Stephanopoulos M. (2000). Low-temperature water-gas shift reaction over Cu- and Ni-loaded cerium oxide catalysts. Appl. Catal. B Environ..

[24] Hori C.E., Permana H., Ng K.Y.S., Brenner A., More K., Rahmoeller K.M., Belton D. (1998). Thermal stability of oxygen storage properties in a mixed CeO_2_-ZrO_2_ system. Appl. Catal. B Environ..

[25] Zaki M.I., Hasan M.A., Al-Sagheer F.A., Pasupulety L. (2001). In situ FTIR spectra of pyridine adsorbed on SiO2–Al2O3, TiO2, ZrO2 and CeO2: General considerations for the identification of acid sites on surfaces of finely divided metal oxides. Colloids Surf. A Physicochem. Eng. Asp..

[26] Trovarelli A., de Leitenburg C., Boaro M., Dolcetti G. (1999). The utilization of ceria in industrial catalysis. Catal. Today.

[27] Tuller H.L. (2000). Ionic conduction in nanocrystalline materials. Solid State Ion..

[28] Abad A., Concepción P., Corma A., García H. (2005). A collaborative effect between gold and a support induces the selective oxidation of alcohols. Angew. Chem. Int. Ed..

[29] Kundakovic L., Flytzani-Stephanopoulos M. (1998). Reduction characteristics of copper oxide in cerium and zirconium oxide systems. Appl. Catal. A Gen..

[30] Wang S., Lu G.Q. (1998). Role of CeO_2_ in Ni/CeO_2_–Al_2_O_3_ catalysts for carbon dioxide reforming of methane. Appl. Catal. B Environ..

[31] Hilaire S., Wang X., Luo T., Gorte R.J., Wagner J. (2001). A comparative study of water-gas-shift reaction over ceria supported metallic catalysts. Appl. Catal. A Gen..

[32] Fu Q., Weber A., Flytzani-Stephanopoulos M. (2001). Nanostructured Au–CeO_2_ Catalysts for Low-Temperature Water–Gas Shift. Catal. Lett..

[33] Rosenzweig A.C. (2015). Biochemistry: Breaking methane. Nature.

[34] Ta N., Liu J., Chenna S., Crozier P.A., Li Y., Chen A., Shen W. (2012). Stabilized Gold Nanoparticles on Ceria Nanorods by Strong Interfacial Anchoring. J. Am. Chem. Soc..

[35] Han Z.-K., Zhang L., Liu M., Ganduglia-Pirovano M.V., Gao Y. (2019). The Structure of Oxygen Vacancies in the Near-Surface of Reduced CeO_2_ (111) Under Strain. Front. Chem..

[36] Mai H.X., Sun L.D., Zhang Y.W., Si R., Feng W., Zhang H.P., Liu H.C., Yan C.H. (2005). Shape-selective synthesis and oxygen storage behavior of ceria nanopolyhedra, nanorods, and nanocubes. J. Phys. Chem. B.

[37] Skorodumova N.V., Simak S.I., Lundqvist B.I., Abrikosov I.A., Johansson B. (2002). Quantum origin of the oxygen storage capability of ceria. Phys. Rev. Lett..

[38] Zhou K., Wang X., Sun X., Peng Q., Li Y. (2005). Enhanced catalytic activity of ceria nanorods from well-defined reactive crystal planes. J. Catal..

[39] Limbach L.K., Li Y., Grass R.N., Brunner T.J., Hintermann M.A., Muller M., Gunther D., Stark W.J. (2005). Oxide nanoparticle uptake in human lung fibroblasts: Effects of particle size, agglomeration, and diffusion at low concentrations. Environ. Sci. Technol..

[40] Tarnuzzer R.W., Colon J., Patil S., Seal S. (2005). Vacancy engineered ceria nanostructures for protection from radiation-induced cellular damage. Nano Lett..

[41] Chen D., Ran R., Zhang K., Wang J., Shao Z. (2009). Intermediate-temperature electrochemical performance of a polycrystalline PrBaCo2O5+δ cathode on samarium-doped ceria electrolyte. J. Power Sources.

[42] Sharma S., Pollet B.G. (2012). Support materials for PEMFC and DMFC electrocatalysts—A review. J. Power Sources.

[43] Andersson D.A., Simak S.I., Skorodumova N.V., Abrikosov I.A., Johansson B. (2006). Optimization of ionic conductivity in doped ceria. Proc. Natl. Acad. Sci. USA.

[44] Chuayboon S., Abanades S., Rodat S. (2019). Syngas production via solar-driven chemical looping methane reforming from redox cycling of ceria porous foam in a volumetric solar reactor. Chem. Eng. J..

[45] Bruix A., Rodriguez J.A., Ramírez P.J., Senanayake S.D., Evans J., Park J.B., Stacchiola D., Liu P., Hrbek J., Illas F. (2012). A new type of strong metal-support interaction and the production of H2 through the transformation of water on Pt/CeO2(111) and Pt/CeO(x)/TiO2(110) catalysts. J. Am. Chem. Soc..

[46] Ratnasamy C., Wagner J.P. (2009). Water Gas Shift Catalysis. Catal. Rev..

[47] Farmer J.A., Campbell C.T. (2010). Ceria Maintains Smaller Metal Catalyst Particles by Strong Metal-Support Bonding. Science.

[48] Wang X., Rodriguez J.A., Hanson J.C., Gamarra D., Martínez-Arias A., Fernández-García M. (2006). In Situ Studies of the Active Sites for the Water Gas Shift Reaction over Cu−CeO_2_ Catalysts:  Complex Interaction between Metallic Copper and Oxygen Vacancies of Ceria. J. Phys. Chem. B.

[49] Sharma S., Hu Z., Zhang P., McFarland E.W., Metiu H. (2011). CO_2_ methanation on Ru-doped ceria. J. Catal..

[50] Wu Z., Jin R., Wang H., Liu Y. (2009). Effect of ceria doping on SO_2_ resistance of Mn/TiO_2_ for selective catalytic reduction of NO with NH3 at low temperature. Catal. Commun..

[51] Roy T., Sahani S., Chandra Sharma Y. (2020). Study on kinetics-thermodynamics and environmental parameter of biodiesel production from waste cooking oil and castor oil using potassium modified ceria oxide catalyst. J. Clean. Prod..

[52] Shapovalov V., Metiu H. (2007). Catalysis by doped oxides: CO oxidation by AuxCe1−xO_2_. J. Catal..

[53] Camellone M.F., Fabris S. (2009). Reaction Mechanisms for the CO Oxidation on Au/CeO2 Catalysts: Activity of Substitutional Au3+/Au+ Cations and Deactivation of Supported Au+ Adatoms. J. Am. Chem. Soc..

[54] Aneggi E., Boaro M., de Leitenburg C., Dolcetti G., Trovarelli A. (2006). Insights into the redox properties of ceria-based oxides and their implications in catalysis. J. Alloy. Compd..

[55] Vivier L., Duprez D. (2010). Ceria-based solid catalysts for organic chemistry. ChemSusChem.

[56] Tana, Zhang M., Li J., Li H., Li Y., Shen W. (2009). Morphology-dependent redox and catalytic properties of CeO2 nanostructures: Nanowires, nanorods and nanoparticles. Catal. Today.

[57] Gorte R.J. (2010). Ceria in catalysis: From automotive applications to the water–gas shift reaction. AIChE J..

[58] Zhong L.-S., Hu J.-S., Cao A.-M., Liu Q., Song W.-G., Wan L.-J. (2007). 3D Flowerlike Ceria Micro/Nanocomposite Structure and Its Application for Water Treatment and CO Removal. Chem. Mater..

[59] Harris M.E., Hensley K., Butterfield D.A., Leedle R.A., Carney J.M. (1995). Direct evidence of oxidative injury produced by the Alzheimer’s β-Amyloid peptide (1–40) in cultured hippocampal neurons. Exp. Neurol..

[60] Lanone S., Rogerieux F., Geys J., Dupont A., Maillot-Marechal E., Boczkowski J., Lacroix G., Hoet P. (2009). Comparative toxicity of 24 manufactured nanoparticles in human alveolar epithelial and macrophage cell lines. Part. Fibre Toxicol..

[61] Lykaki M., Pachatouridou E., Carabineiro S.A.C., Iliopoulou E., Andriopoulou C., Kallithrakas-Kontos N., Boghosian S., Konsolakis M. (2018). Ceria nanoparticles shape effects on the structural defects and surface chemistry: Implications in CO oxidation by Cu/CeO2 catalysts. Appl. Catal. B Environ..

[62] Pagliari F., Mandoli C., Forte G., Magnani E., Pagliari S., Nardone G., Licoccia S., Minieri M., Di Nardo P., Traversa E. (2012). Cerium Oxide Nanoparticles Protect Cardiac Progenitor Cells from Oxidative Stress. ACS Nano.

[63] Ornatska M., Sharpe E., Andreescu D., Andreescu S. (2011). Paper Bioassay Based on Ceria Nanoparticles as Colorimetric Probes. Anal. Chem..

[64] Das M., Patil S., Bhargava N., Kang J.-F., Riedel L.M., Seal S., Hickman J.J. (2007). Auto-catalytic ceria nanoparticles offer neuroprotection to adult rat spinal cord neurons. Biomaterials.

[65] Korsvik C., Patil S., Seal S., Self W.T. (2007). Superoxide dismutase mimetic properties exhibited by vacancy engineered ceria nanoparticles. Chem. Commun..

[66] Mahmoudi M., Sant S., Wang B., Laurent S., Sen T. (2011). Superparamagnetic iron oxide nanoparticles (SPIONs): Development, surface modification and applications in chemotherapy. Adv. Drug Deliv. Rev..

[67] Carofiglio M., Barui S., Cauda V., Laurenti M. (2020). Doped Zinc Oxide Nanoparticles: Synthesis, Characterization and Potential Use in Nanomedicine. Appl. Sci..

[68] Asati A., Santra S., Kaittanis C., Perez J.M. (2010). Surface-Charge-Dependent Cell Localization and Cytotoxicity of Cerium Oxide Nanoparticles. ACS Nano.

[69] Asati A., Santra S., Kaittanis C., Nath S., Perez J.M. (2009). Oxidase-like activity of polymer-coated cerium oxide nanoparticles. Angew. Chem..

[70] Perez J.M., Asati A., Nath S., Kaittanis C. (2008). Synthesis of biocompatible dextran-coated nanoceria with pH-dependent antioxidant properties. Small.

[71] Kim C.K., Kim T., Choi I.-Y., Soh M., Kim D., Kim Y.-J., Jang H., Yang H.-S., Kim J.Y., Park H.-K. (2012). Ceria Nanoparticles that can Protect against Ischemic Stroke. Angew. Chem. Int. Ed..

[72] Schubert D., Dargusch R., Raitano J., Chan S.W. (2006). Cerium and yttrium oxide nanoparticles are neuroprotective. Biochem. Biophys. Res. Commun..

[73] Park E.-J., Choi J., Park Y.-K., Park K. (2008). Oxidative stress induced by cerium oxide nanoparticles in cultured BEAS-2B cells. Toxicology.

[74] Zhang P., Ma Y., Zhang Z., He X., Zhang J., Guo Z., Tai R., Zhao Y., Chai Z. (2012). Biotransformation of Ceria Nanoparticles in Cucumber Plants. ACS Nano.

[75] Ali D., Alarifi S., Alkahtani S., AlKahtane A.A., Almalik A. (2015). Cerium Oxide Nanoparticles Induce Oxidative Stress and Genotoxicity in Human Skin Melanoma Cells. Cell Biochem. Biophys..

[76] Alili L., Sack M., Karakoti A.S., Teuber S., Puschmann K., Hirst S.M., Reilly C.M., Zanger K., Stahl W., Das S. (2011). Combined cytotoxic and anti-invasive properties of redox-active nanoparticles in tumor-stroma interactions. Biomaterials.

[77] Ristow M. (2006). Oxidative metabolism in cancer growth. Curr. Opin. Clin. Nutr. Metab. Care.

[78] Singh S., Kumar U., Gittess D., Sakthivel T.S., Babu B., Seal S. (2021). Cerium oxide nanomaterial with dual antioxidative scavenging potential: Synthesis and characterization. J. Biomater. Appl..

[79] Celardo I., Traversa E., Ghibelli L. (2011). Cerium oxide nanoparticles: A promise for applications in therapy. J. Exp. Ther. Oncol..

[80] Kunwar D., Zhou S., DeLaRiva A., Peterson E.J., Xiong H., Pereira-Hernández X.I., Purdy S.C., ter Veen R., Brongersma H.H., Miller J.T. (2019). Stabilizing High Metal Loadings of Thermally Stable Platinum Single Atoms on an Industrial Catalyst Support. ACS Catal..

[81] Pastor-Pérez L., Patel V., Le Saché E., Reina T.R. (2020). CO_2_ methanation in the presence of methane: Catalysts design and effect of methane concentration in the reaction mixture. J. Energy Inst..

[82] Ding Y., Wu Q., Lin B., Guo Y., Guo Y., Wang Y., Wang L., Zhan W. (2020). Superior catalytic activity of a Pd catalyst in methane combustion by fine-tuning the phase of ceria-zirconia support. Appl. Catal. B Environ..

[83] Maria Magdalane C., Kaviyarasu K., Matinise N., Mayedwa N., Mongwaketsi N., Letsholathebe D., Mola G.T., AbdullahAl-Dhabi N., Arasu M.V., Henini M. (2018). Evaluation on La_2_O_3_ garlanded ceria heterostructured binary metal oxide nanoplates for UV/visible light induced removal of organic dye from urban wastewater. S. Afr. J. Chem. Eng..

[84] Jones J., Xiong H., DeLaRiva A.T., Peterson E.J., Pham H., Challa S.R., Qi G., Oh S., Wiebenga M.H., Pereira Hernández X.I. (2016). Thermally stable single-atom platinum-on-ceria catalysts via atom trapping. Science.

[85] Das S., Ashok J., Bian Z., Dewangan N., Wai M.H., Du Y., Borgna A., Hidajat K., Kawi S. (2018). Silica–Ceria sandwiched Ni core–shell catalyst for low temperature dry reforming of biogas: Coke resistance and mechanistic insights. Appl. Catal. B Environ..

[86] Liu Q., Yang Y., Lv X., Ding Y., Zhang Y., Jing J., Xu C. (2017). One-step synthesis of uniform nanoparticles of porphyrin functionalized ceria with promising peroxidase mimetics for H_2_O_2_ and glucose colorimetric detection. Sens. Actuators B Chem..

[87] Sun L., Ding Y., Jiang Y., Liu Q. (2017). Montmorillonite-loaded ceria nanocomposites with superior peroxidase-like activity for rapid colorimetric detection of H2O2. Sens. Actuators B Chem..

[88] Atif M., Iqbal S., Fakhar-E-Alam M., Ismail M., Mansoor Q., Mughal L., Aziz M.H., Hanif A., Farooq W.A. (2019). Manganese-Doped Cerium Oxide Nanocomposite Induced Photodynamic Therapy in MCF-7 Cancer Cells and Antibacterial Activity. BioMed Res. Int..

[89] Pelletier D.A., Suresh A.K., Holton G.A., McKeown C.K., Wang W., Gu B., Mortensen N.P., Allison D.P., Joy D.C., Allison M.R. (2010). Effects of Engineered Cerium Oxide Nanoparticles on Bacterial Growth and Viability. Appl. Environ. Microbiol..

[90] Yu H., Jin F., Liu D., Shu G., Wang X., Qi J., Sun M., Yang P., Jiang S., Ying X. (2020). ROS-responsive nano-drug delivery system combining mitochondria-targeting ceria nanoparticles with atorvastatin for acute kidney injury. Theranostics.

[91] He L., Huang G., Liu H., Sang C., Liu X., Chen T. (2020). Highly bioactive zeolitic imidazolate framework-8–capped nanotherapeutics for efficient reversal of reperfusion-induced injury in ischemic stroke. Sci. Adv..

[92] Kwon H.J., Cha M.-Y., Kim D., Kim D.K., Soh M., Shin K., Hyeon T., Mook-Jung I. (2016). Mitochondria-Targeting Ceria Nanoparticles as Antioxidants for Alzheimer’s Disease. ACS Nano.

[93] Aksnes D.W., Langfeldt L., Wouters P. (2019). Citations, Citation Indicators, and Research Quality: An Overview of Basic Concepts and Theories. SAGE Open.

[94] Ebrahimi P., Kumar A., Khraisheh M. (2020). A review of recent advances in water-gas shift catalysis for hydrogen production. Emergent Mater..

[95] Crabtree G.W., Dresselhaus M.S. (2008). The Hydrogen Fuel Alternative. MRS Bull..

[96] Nyoka M., Choonara Y.E., Kumar P., Kondiah P.P.D., Pillay V. (2020). Synthesis of Cerium Oxide Nanoparticles Using Various Methods: Implications for Biomedical Applications. Nanomaterials.

[97] Li M., Liu Z., Hu Y., Wang M., Li H. (2008). Effect of doping elements on catalytic performance of CeO_2_-ZrO_2_ solid solutions. J. Rare Earths.

[98] Mousavi-Kamazani M., Azizi F. (2019). Facile sonochemical synthesis of Cu doped CeO_2_ nanostructures as a novel dual-functional photocatalytic adsorbent. Ultrason. Sonochem..

[99] Mužina K., Kurajica S., Dražić G., Guggenberger P., Matijašić G. (2021). True doping levels in hydrothermally derived copper-doped ceria. J. Nanopart. Res..

[100] Sharma P.K., Pandey O.P. (2022). Enhanced photocatalytic activity with metal ion doping and co-doping in CeO_2_ nanoparticles. Solid State Sci..

[101] Walkey C., Das S., Seal S., Erlichman J., Heckman K., Ghibelli L., Traversa E., McGinnis J.F., Self W.T. (2015). Catalytic Properties and Biomedical Applications of Cerium Oxide Nanoparticles. Environ. Sci. Nano.

[102] Ighodaro O.M., Akinloye O.A. (2018). First line defence antioxidants-superoxide dismutase (SOD), catalase (CAT) and glutathione peroxidase (GPX): Their fundamental role in the entire antioxidant defence grid. Alex. J. Med..

[103] Nourmohammadi E., Khoshdel-sarkarizi H., Nedaeinia R., Darroudi M., Kazemi Oskuee R. (2020). Cerium oxide nanoparticles: A promising tool for the treatment of fibrosarcoma in-vivo. Mater. Sci. Eng. C.

[104] Alpaslan E., Yazici H., Golshan N., Ziemer K., Webster T. (2015). Dextran coated cerium oxide nanoparticles for inhibiting bone cancer cell functions. Biomater. Sci. Process. Prop. Appl. V.

[105] Valdeperez D., Wang T., Eußner J.P., Weinert B., Hao J., Parak W.J., Dehnen S., Pelaz B. (2017). Polymer-coated nanoparticles: Carrier platforms for hydrophobic water- and air-sensitive metallo-organic compounds. Pharmacol. Res..

[106] Bagheri S., Khalil M., Muhd Julkapli N. (2020). Cerium (IV) oxide nanocomposites: Catalytic properties and industrial application. J. Rare Earth..

[107] Fu Q., Deng W., Saltsburg H., Flytzani-Stephanopoulos M. (2005). Activity and stability of low-content gold–cerium oxide catalysts for the water–gas shift reaction. Appl. Catal. B Environ..

[108] Alpaslan E., Yazici H., Golshan N.H., Ziemer K.S., Webster T.J. (2015). pH-Dependent Activity of Dextran-Coated Cerium Oxide Nanoparticles on Prohibiting Osteosarcoma Cell Proliferation. ACS Biomater. Sci. Eng..

[109] Datta A., Mishra S., Manna K., Saha K.D., Mukherjee S., Roy S. (2020). Pro-Oxidant Therapeutic Activities of Cerium Oxide Nanoparticles in Colorectal Carcinoma Cells. ACS Omega.

[110] Alpaslan E., Geilich B.M., Yazici H., Webster T.J. (2017). pH-Controlled Cerium Oxide Nanoparticle Inhibition of Both Gram-Positive and Gram-Negative Bacteria Growth. Sci. Rep..

[111] Chelombitko M.A. (2018). Role of Reactive Oxygen Species in Inflammation: A Minireview. Mosc. Univ. Biol. Sci. Bull..

[112] Dewberry L.K., Zgheib C., Hilton S.A., Seal S., Newsom J., Krebs M.D., Hu J., Xu J., Liechty K.W. (2019). Cerium Oxide Nanoparticle-miR146a Decreases Inflammation in a Murine Dextran Sodium Sulfate Colitis Model. J. Am. Coll. Surg..

[113] Chai W.F., Tang K.S. (2021). Protective potential of cerium oxide nanoparticles in diabetes mellitus. J. Trace Elem. Med. Biol..

[114] Wei F., Neal C.J., Sakthivel T.S., Kean T., Seal S., Coathup M.J. (2021). Multi-functional cerium oxide nanoparticles regulate inflammation and enhance osteogenesis. Mater. Sci. Eng. C.

[115] Jan A., Shin J., Ahn J., Yang S., Yoon K.J., Son J.-W., Kim H., Lee J.-H., Ji H.-I. (2019). Promotion of Pt/CeO2 catalyst by hydrogen treatment for low-temperature CO oxidation. RSC Adv..

[116] Muravev V., Spezzati G., Su Y.-Q., Parastaev A., Chiang F.-K., Longo A., Escudero C., Kosinov N., Hensen E.J.M. (2021). Interface dynamics of Pd–CeO2 single-atom catalysts during CO oxidation. Nat. Catal..

[117] Sack Z., Bader S., Brenneisen P. (2017). Cerium Oxide Nanoparticles as Novel Tool in Glioma Treatment: An In vitro Study. J. Nanomed. Nanotechnol..

[118] Naz S., Beach J., Heckert B., Tummala T., Pashchenko O., Banerjee T., Santra S. (2017). Cerium oxide nanoparticles: A ‘radical’ approach to neurodegenerative disease treatment. Nanomedicine.

[119] Gunawan C., Lord M.S., Lovell E., Wong R.J., Jung M.S., Oscar D., Mann R., Amal R. (2019). Oxygen-Vacancy Engineering of Cerium-Oxide Nanoparticles for Antioxidant Activity. ACS Omega.

[120] Gella A., Durany N. (2009). Oxidative stress in Alzheimer disease. Cell Adhes. Migr..

[121] Heneka M.T., Carson M.J., El Khoury J., Landreth G.E., Brosseron F., Feinstein D.L., Jacobs A.H., Wyss-Coray T., Vitorica J., Ransohoff R.M. (2015). Neuroinflammation in Alzheimer’s disease. Lancet Neurol..

[122] D’Angelo B., Santucci S., Benedetti E., Di Loreto S., Phani A., Falone S., Amicarelli F., Cerù M., Cimini A. (2009). Cerium Oxide Nanoparticles Trigger Neuronal Survival in a Human Alzheimer Disease Model By Modulating BDNF Pathway. Curr. Nanosci..

[123] Siposova K., Huntosova V., Shlapa Y., Lenkavska L., Macajova M., Belous A., Musatov A. (2019). Advances in the Study of Cerium Oxide Nanoparticles: New Insights into Antiamyloidogenic Activity. ACS Appl. Bio Mater..

[124] Kumari M., Singh S.P., Chinde S., Rahman M.F., Mahboob M., Grover P. (2014). Toxicity Study of Cerium Oxide Nanoparticles in Human Neuroblastoma Cells. Int. J. Toxicol..

[125] Lin W., Huang Y.-W., Zhou X.-D., Ma Y. (2006). Toxicity of Cerium Oxide Nanoparticles in Human Lung Cancer Cells. Int. J. Toxicol..

[126] Niu J., Azfer A., Rogers L.M., Wang X., Kolattukudy P.E. (2007). Cardioprotective effects of cerium oxide nanoparticles in a transgenic murine model of cardiomyopathy. Cardiovasc. Res..

